# *Rosa canina* Extract Attenuates Muscle Atrophy in L6 Myotubes and Immobilized Mice

**DOI:** 10.3390/nu17213462

**Published:** 2025-11-02

**Authors:** Hyerin Lee, Mi-Bo Kim, Junhui Kang, Jae-Kwan Hwang, Bohkyung Kim

**Affiliations:** 1 Graduate Program in Bioindustrial Engineering, College of Life Science and Biotechnology, Yonsei University, Seoul 03722, Republic of Korea; hshs7915@naver.com (H.L.); junrim2577@naver.com (J.K.); 2 Department of Food Science and Nutrition, Pukyong National University, Busan 48513, Republic of Korea; mibokim1120@gmail.com; 3 Department of Food Science and Nutrition, Pusan National University, Busan 46241, Republic of Korea; 4 BK21 FOUR Program: Precision Nutrition Program for Future Global Leaders, Pusan National University, Busan 46241, Republic of Korea

**Keywords:** *Rosa canina*, rosehip, muscle atrophy, L6 myotubes, immobilization

## Abstract

**Background**: Skeletal muscle is essential not only for structural integrity but also metabolic homeostasis. Muscle atrophy, the loss of muscle mass and function, is closely linked to chronic and metabolic disorders and is driven by chronic inflammation, oxidative stress, impaired myogenesis, and disrupted protein homeostasis. The present study aimed to evaluate the protective effects and underlying mechanisms of *Rosa canina* extract (RCE), a polyphenol-rich plant known for its antioxidant and anti-inflammatory properties, in vitro and in vivo models of muscle atrophy. **Methods**: We investigated the effects of RCE in TNF-α-treated L6 myotubes and a mouse model (eight-week-old male C57BL/6N) of immobilization-induced muscle atrophy. Markers of inflammation, oxidative stress, myogenesis, protein turnover, and anabolic signaling were analyzed via RT-PCR, Western blotting and ELISA. Muscle mass, performance, micro-CT imaging, and histological cross-sectional area were assessed in vivo. **Results**: RCE suppressed pro-inflammatory cytokines, restored antioxidant enzyme expression, and preserved myogenic markers. It inhibited muscle proteolysis by downregulating the genes involved in protein degradation and promoted protein synthesis by via activation of the PI3K/Akt/mTOR pathway. In mice, RCE mitigated muscle mass loss, preserved fiber cross-sectional area, improved strength and endurance, and restored muscle volume. **Conclusions**: RCE attenuated muscle atrophy by targeting inflammation, oxidative stress, proteolysis, and impaired anabolism. These findings highlight RCE as a promising natural therapeutic for preserving muscle health and metabolic homeostasis.

## 1. Introduction

Skeletal muscle is a dynamic and essential tissue that comprises approximately 40% of the body weight, accounts for 50–70% of the body protein, and is responsible for 30–50% of whole-body protein turnover [1]. It plays a crucial role in performing locomotor and postural functions and is integral to physical activities and organic metabolism [2]. Given its central role in energy homeostasis, maintaining the mass of skeletal muscle is vital for supporting the physiological demands of various organs and preventing metabolic disorders [3]. Therefore, understanding the mechanisms that govern muscle health is crucial, particularly in the context of muscle atrophy, the pathological reduction of muscle mass and function. Muscle atrophy is a common pathological feature of several chronic conditions, including aging, sarcopenia, cancer cachexia, malnutrition and inflammatory diseases [4]. One of the primary drivers of muscle atrophy is chronic inflammation, often mediated by pro-inflammatory cytokines such as tumor necrosis factor-alpha (TNF-α) and interleukin-6 (IL-6) [5]. Inflammation plays a significant role in the development of muscle atrophy, particularly through the action of pro-inflammatory cytokines, which are often elevated in conditions like cachexia, sepsis, and immobilization [6]. Systemic inflammation can activate and upregulate proteolysis, particularly through the ubiquitin-proteasome and lysosomal degradation pathways. Furthermore, inflammatory signaling suppresses anabolic pathways, including the insulin-like growth factor 1 (IGF-1)/phosphatidylinositol 3-kinase (PI3K)/protein kinase B (Akt)/mammalian target of rapamycin (mTOR) axis, thereby reducing protein synthesis [5,6,7]. Oxidative stress plays a crucial role in the pathogenesis of muscle atrophy. Excessive production of reactive oxygen species (ROS) leads to cellular damage, mitochondrial dysfunction, and activation of redox-sensitive transcription factors such as nuclear factor kappa B (NF-κB) and forkhead box O3a (FoxO3a), which can promote muscle proteolysis [8,9]. Oxidative damage also interferes with myogenic signaling and regeneration, compounding the decline in muscle mass and quality [9,10]. The convergence of inflammation, oxidative stress, enhanced protein degradation, and impaired protein synthesis creates a detrimental cycle that accelerates muscle atrophy. Given the complex interplay of inflammation, oxidative stress, and altered protein turnover in muscle atrophy, natural products with anti-inflammatory and antioxidant effects have been increasingly recognized for their potential in preventing or mitigating muscle atrophy.

*Rosa canina*, commonly known as rosehip, is a plant belonging to the Rosaceae family that has been used as a traditional herb to treat various symptoms, including inflammation, colds, flu, ulcers, and chronic pain, in Europe, North America, and Asia [11]. *Rosa canina* exhibited various biological activities, including antioxidant, anti-osteoarthritis, anticancer, antidiabetic, and anti-obesity effects [12]. Studies have demonstrated that *Rosa canina* extracts can effectively scavenge free radicals, reduce oxidative damage, and suppress the production of pro-inflammatory cytokines in various in vitro and in vivo models of inflammation and oxidative stress [13,14,15,16,17]. Furthermore, its polyphenolic components have been shown to influence signaling pathways involved in protein synthesis and degradation. Despite these promising biological activities, the direct effects of *Rosa canina* on muscle atrophy have been underexplored. Therefore, this study aims to elucidate the molecular mechanisms by which *Rosa canina* modulates key pathways involved in muscle atrophy induced by L6 myotubes and immobilized C57BL/6N mice.

## 2. Materials and Methods

### 2.1. Preparation of Rosa canina Extract (RCE)

The dried fruits of *Rosa canina*, provided by Phytomedi Inc. (Seoul, Republic of Korea), were extracted with water at 60 °C for 4 h, and then the extract was filtered using Whatman grade 2 qualitative filter paper (Whatman, Maidstone, UK). The water in the extract was evaporated using a rotary vacuum evaporator (Heidolph Instruments GmbH & Co. KG, Schwabach, Germany). The yield of RCE was 53.5% (*w*/*w*).

### 2.2. Chemical Reagents

Recombinant rat TNF-α was purchased from PeproTech (Rocky Hill, NJ, USA). Antibodies against FoxO3a, phosphorylated FoxO3a (p-FoxO3a), PI3K, phosphorylated-PI3K (p-PI3K), Akt, phosphorylated-Akt (p-Akt), mTOR, phosphorylated-mTOR (p-mTOR), 70-kDa ribosomal S6 kinase (p70S6K), phosphorylated-p70S6K (p-p70S6K), 4E-binding protein 1 (4EBP1), phosphorylated-4EBP1 (p-4EBP1), and α-tubulin were purchased from Cell Signaling Technology (Beverly, MA, USA), and NF-κB was purchased from Santa Cruz Biotechnology Inc. (Santa Cruz, CA, USA). Horseradish peroxidase-linked secondary antibodies were obtained from Bethyl Laboratories Inc. (Montgomery, TX, USA).

### 2.3. L6 Myoblast Culture and Differentiation

L6 myoblast cells were purchased from the American Type Culture Collection (ATCC; Manassas, VA, USA). Cells were maintained in Dulbecco’s modified Eagle’s medium (DMEM; Hyclone, Logan, UT, USA) with 10% fetal bovine serum (FBS; Hyclone) and 1% P/S (100 IU penicillin A plus 100 μg/mL streptomycin) at 37 °C in a 5% CO_2_ humidified atmosphere. When the cell reached approximately 70–80% confluence, the culture medium was exchanged with differentiation medium consisting of DMEM supplemented with 1% horse serum (Gibco, Gaithersburg, MD, USA), 1% FBS, and 1% P/S to induce differentiation into myotubes. The differentiation medium was refreshed every other day for a total of 6 days. Fully differentiated L6 myotubes were treated with 50 ng/mL recombinant rat TNF-α to induce skeletal muscle atrophy, either in the presence or absence of RCE at concentrations of 40 and 80 µg/mL for 24 h.

### 2.4. Cell Viability

Cell viability was evaluated using the thiazolyl blue tetrazolium bromide (MTT) assay, as described in a previous study [18]. Briefly, L6 myoblasts were treated with various concentrations (0–100 μg/mL) of RCE for 24 h, followed by incubation with MTT solution (0.5 mg/mL) at 37 °C for 3 h. The resulting formazan crystals were dissolved in dimethyl sulfoxide, and absorbance was measured at 540 nm using a microplate reader (VERSAmax, Molecular Devices, Sunnyvale, CA, USA).

### 2.5. Animal Study

Eight-week-old male C57BL/6N mice were obtained from Samtako (Gyeonggi, Republic of Korea). They were housed under controlled conditions (23 ± 2 °C, 55 ± 5% relative humidity) with a 12 h light/12 h dark cycle at the Yonsei Laboratory Animal Research Center (YLARC; Seoul, Republic of Korea). Following a 1-week acclimatization period, a total of 40 mice were randomly assigned to four groups (*n* = 10 per group). Group I (CON) received no surgery and was supplemented with saline. Group II (IMM) underwent immobilization surgery and was supplemented with saline. Groups III and IV (RCL) and (RCH) received the surgery and were orally administered RCE at 200 and 400 mg/kg/day, respectively. The sample size was calculated using G*Power 3.1.9.4 for one-way ANOVA (f = 0.59, α = 0.05, power = 0.80), yielding 36 mice (9 per group). To account for potential failure, one additional animal was included per group, resulting in a total of 40 mice (10 per group). Muscle atrophy was induced via immobilization surgery as previously described [19]. Briefly, mice were anesthetized intraperitoneally with 325 mg/kg tribromoethanol (Sigma-Aldrich, St. Louis, MO, USA), and the right hindlimb was immobilized using the Acos 35W skin stapler (Sunmedix Co., Ltd., Gyeonggi, Republic of Korea) by inserting one tine into the foot and another into the upper limb. After 1 week of immobilization, the staple was removed under the same anesthesia, and the mice received daily oral administration of saline or RCE for an additional week. Functional assessments, including grip strength and treadmill tests, were measured. At the end of the experiment, mice were anesthetized with 350 mg/kg 2,2,2-tribromoethanol and sacrificed by cardiac puncture. The skeletal muscles, including gastrocnemius (GA), soleus (SOL), tibialis anterior (TA), and extensor digitorum longus (EDL) muscles, were carefully dissected from the right hindlimb, weighed immediately, and snap-frozen in liquid nitrogen. The collected samples were then divided into two portions: one portion was fixed in 10% neutral-buffered formalin for histological evaluation, and the remaining portion was stored at −80 °C for subsequent biochemical analyses. All animal experimental procedures were reviewed and approved by the Institute of Animal Care and Use Committee (IACUC) of Yonsei University (Seoul, Republic of Korea) (Permit No.: IACUC-A-202012-1185-01). Cages were placed alternately among the groups to reduce potential confounding effects, and all experimental treatments and assessments were performed in a consistent order. Blinding was not applied because the study involved daily oral dosing, body weight measurements, and behavioral evaluations that required direct involvement of the investigators. Humane endpoints were not predefined, as no significant pain or distress was expected from the experimental design. The animals’ health condition was routinely monitored during the daily administration period. All procedures were performed in accordance with institutional animal care regulations to minimize discomfort and stress.

### 2.6. Grip Strength Test

The day prior to sacrifice, grip strength was assessed using a grip strength meter (Panlab, Barcelona, Spain) to measure both forelimb and hindlimb muscle strength. The maximum pull force was measured in grams (g) using a digital force converter. Following stabilization, the gauge was reset to 0 g, and each mouse was gently held by the tail and slowly pulled backward by an investigator until it released its grip on the grid. Grip strength force was recorded, with at least five recordings per mouse and the mean values were calculated for statistical analysis.

### 2.7. Treadmill Test

Endurance capacity was evaluated using a rodent treadmill machine (LE8710MTS; Panlab, Barcelona, Spain) by measuring both running time and distance. Mice were subjected to a graded exercise test until they reached complete exhaustion, defined as the inability to escape from an electric shock for 30 s or longer. The treadmill protocol began at a speed of 15 cm/s for 10 min, followed by incremental increases of 1 cm/s every min until a maximum speed of 35 cm/s was reached. Upon reaching the point of exhaustion, the experiment was terminated, and mice were subsequently anesthetized for sacrifice.

### 2.8. Micro-Computed Tomography (Micro-CT) Imaging

Muscle volumes and density were measured using a small animal positron emission tomography (PET)/CT/single photon emission tomography (SPECT) system (Siemens Inveon, Knoxville, TN, USA) at the Center for Evaluation of Biomaterials (Pohang Technopark, Pohang, Republic of Korea). The Inveon Research Workplace software 4.2 (Siemens Inveon) was used for the analysis of CT images.

### 2.9. Histological Analysis

For histological analysis, TA muscles were fixed in 10% formalin solution (Junsei, Tokyo, Japan), dehydrated, and embedded in paraffin blocks. The paraffin blocks were cut at a thickness of 4 μm and stained with hematoxylin & eosin (H&E). The stained sections of each sample were then imaged in randomly selected areas by a CK40 inverted microscope (Olympus, Tokyo, Japan) equipped with a T500 camera (eXcope, Daejeon, Republic of Korea). The cross-sectional area (CSA) of muscle fibers was quantified using the ImageJ software 1.47 (National Institutes of Health, Bethesda, MD, USA), and the mean values were calculated for statistical analysis.

### 2.10. Enzyme-Linked Immunosorbent Assay (ELISA)

Serum was obtained by centrifuging blood samples at 1300× *g* for 15 min. IL-15 levels in serum were quantified using a commercially available ELISA kit (Merck Millipore, Burlington, MA, USA) following the manufacturer’s instructions.

### 2.11. Reverse Transcription-Polymerase Chain Reaction (RT-PCR)

Total RNAs were isolated from L6 myotubes and TA muscle tissues using TRIzol reagent (Takara Bio, Otsu, Japan) according to the manufacturer’s instructions. The concentration of isolated total RNA was quantified using the NanoDrop Lite spectrophotometer (Thermo Fisher Scientific Inc., Waltham, MA, USA). The RNA was reverse-transcribed into complementary DNA (cDNA) using the RT-Premix (ELPIS Biotech, Daejeon, Republic of Korea) at 42 °C for 1 h and 95 °C for 5 min. The quantitative polymerase chain reaction amplification was performed with the synthesized cDNA using the SafeDry Taq PCR premix (CellSafe, Gyeonggi, Republic of Korea), and specific primers (Bioneer, Daejeon, Republic of Korea) were implemented by the following process: enzyme activation at 95 °C for 5 min, denaturation at 95 °C for 30 s, annealing at 58–60 °C for 30 s, extension at 72 °C for 45 s, and a final extension at 72 °C for 5 min. The final PCR amplicons were stained with 5X Loading STAR dye (DyneBio, Seongnam, Republic of Korea) and separated by electrophoresis using 1.5% agarose gel. The PCR product band was visualized using the G:BOX imaging analysis system and GeneSys software (https://www.genesys.com/en-sg accessed on (30 October 2025)) (Syngene, Bangalore, India), and quantified using ImageJ software 1.47 (National Institutes of Health).

### 2.12. Western Blot Analysis

L6 myotubes and TA muscle tissues were homogenized in NP40 lysis buffer (ELPIS Biotech) containing a proteinase inhibitor cocktail (Sigma-Aldrich). The protein lysates were clarified by centrifugation at 13,000 rpm for 10 min at 4 °C, and the supernatants were used for Western blot analysis. The concentration of protein was determined by the Bradford assay (Bio-Rad Laboratories Inc., Hercules, CA, USA). Equal amounts of protein were separated by sodium dodecyl sulfate-polyacrylamide gel electrophoresis (SDS-PAGE) and transferred onto 0.45 μm nitrocellulose membranes (GE Healthcare, Piscataway, NJ, USA). Membranes were then blocked with 5% skimmed milk in Tris-buffered saline (DyneBio) containing 0.1% Tween 20 (TBS-T) for 30 min at room temperature and subsequently incubated overnight at 4 °C with primary antibodies against PI3K, p-PI3K, Akt, p-Akt, mTOR, p-mTOR, p70S6K, p-p70S6K, 4EBP1, p-4EBP1, NF-κB, and α-tubulin (1:1000 dilution). After washing, the membranes were incubated with horseradish peroxidase-conjugated secondary antibodies diluted 1:5000 for 2 h at 4 °C. The target proteins on the membranes were detected using an enhanced chemiluminescence (ECL) solution and visualized using the G: BOX imaging analysis system and GeneSys software (Syngene). The band intensities were quantified using ImageJ software (National Institutes of Health).

### 2.13. Statistical Analysis

All experimental data were expressed as mean values ± standard error of the mean (SEM), and *p* values less than 0.05 were considered statistically significant. The significant differences between the groups were detected using one-way analysis of variance (ANOVA), followed by Tukey’s multiple comparison test or an unpaired *t*-test, as performed in GraphPad Prism version 10.0 (GraphPad Software, La Jolla, CA, USA). Outliers were identified using the GraphPad outlier calculator before analysis.

## 3. Results

### 3.1. RCE Alleviated the TNF-α-Induced Inflammation and Oxidative Stress in TNF-α-Induced L6 Myotubes

**Figure 1:**
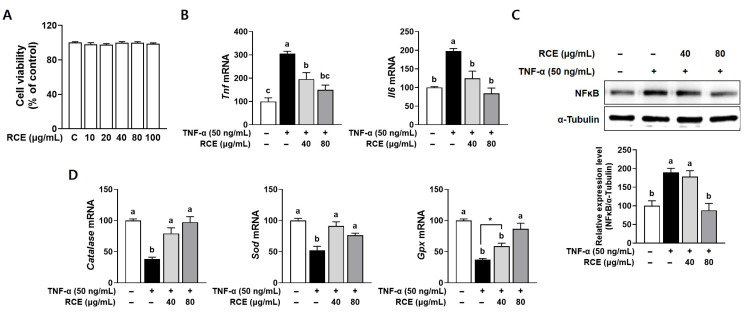
Protective effects of RCE on inflammation and oxidative stress in TNF-α-stimulated L6 myotubes. (**A**) Cell viability was measured by MTT assay. (**B**) The mRNA expression of proinflammatory cytokines was measured by RT-PCR. (**C**) A representative blot of NF-κB protein levels was evaluated by Western blot. α-Tubulin-served as a loading control. (**D**) The mRNA expression of antioxidant enzymes was measured by RT-PCR. Group differences were assessed using one-way analysis of variance (ANOVA). Bars with different letters are significantly different (*p* < 0.05). Asterisk (*) indicates statistically significant differences between the two groups based on unpaired *t*-test results (*p*  <  0.05). Values are means ± SEM. RCE, *Rosa canina* extract; TNF-α, tumor necrosis factor-alpha; *Il6*, gene name of interleukin 6; NFκB, nuclear factor kappa B; *Sod*, gene name of superoxide dismutase; *Gpx*, gene name of glutathione peroxidase.

Chronic inflammation and oxidative stress are closely associated with muscle atrophy, as they synergistically contribute to the dysregulation of muscle protein homeostasis [20]. To evaluate the protective effects of RCE on inflammatory response and oxidative stress, the genes involved in inflammatory signaling and antioxidant defense mechanisms were investigated in TNF-α-stimulated L6 myotubes. RCE treatment at concentrations ranging from 0 to 100 μg/mL showed no significant differences in L6 myotube viability (Figure 1A). Therefore, concentrations below 100 μg/mL were selected for subsequent experiments. The mRNA levels of inflammatory cytokines, including *Tnf* and *Il6*, were significantly upregulated in TNF-α-treated cells. Treatment with RCE markedly downregulated the expression of these cytokines (Figure 1B). In line with these results, a marked induction of NF-κB proteins was observed in TNF-α-stimulated L6 cells, whereas 80 µg/mL of RCE significantly alleviated this increase (Figure 1C). Furthermore, the mRNA abundance of antioxidant enzymes, including *catalase*, superoxide dismutase (*Sod*), and glutathione peroxidase (*Gpx*), was significantly reduced in TNF-α-treated myotubes. Notably, RCE treatment restored the expression of antioxidant genes to near control levels (Figure 1D). These results suggest that RCE might attenuate muscle atrophy by alleviating the inflammatory response and suppressing ROS production.

### 3.2. RCE Prevented the Muscle Protein Degradation Pathway in TNF-α-Induced L6 Myotubes

**Figure 2:**
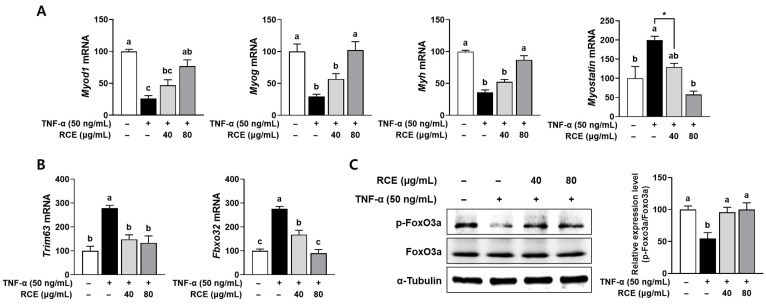
Effects of RCE on myogenesis and protein degradation-related pathway in TNF-α-stimulated L6 myotubes. (**A**) The mRNA expression of genes involved in myogenesis. (**B**) The mRNA expression of genes involved in muscle-specific E3 ubiquitin ligases. The mRNA expressions were measured by RT-PCR. (**C**) The protein levels of phosphorylated and total FoxO3a were assessed using Western blot analysis. α-Tubulin-served as a loading control. Group differences were assessed using one-way analysis of variance (ANOVA). Bars with different letters are significantly different (*p* < 0.05). Asterisk (*) indicates statistically significant differences between the two groups based on unpaired *t*-test results (*p*  <  0.05). Values are means ± SEM. RCE, *Rosa canina* extract; TNF-α, tumor necrosis factor-alpha; *Myog*, gene name of myogenin; *Myh*, gene name of myosin heavy chain; *Trim63*, gene name of muscle ring finger 1; *Fbxo32*, gene name of muscle atrophy F-box; FoxO3a, forkhead box O3a; p-FoxO3a, phosphorylated FoxO3a.

Myogenesis, the process by which muscle fibers are formed, plays a crucial role in the maintenance, regeneration, and repair of muscle tissue. Impaired myogenesis can lead to reduced muscle formation that can contribute to muscle atrophy [21]. Muscle atrophy is primarily attributed to a disruption in the dynamic balance between muscle protein synthesis and degradation [22]. We measured the effects of RCE on myogenesis and proteolysis in L6 cells treated with TNF-α. The mRNA expressions of genes involved in myogenesis, i.e., myogenic differentiation 1 (MyoD, gene name *Myod1*), myogenin (gene name *Myog*), and myosin heavy chain (MHC, gene name *Myh*), were significantly decreased by TNF-α treatment, indicating that TNF-α induces atrophic conditions. The decreased myogenic gene expression was significantly restored by treatment with 80 μg/mL of RCE. Under atrophic conditions in L6 cells, the increased expression of myostatin, the negative regulator of skeletal muscle mass, was attenuated in RCE-treated cells (Figure 2A). The increased expression of the canonical E3 ubiquitin ligases in muscle is a hallmark of muscle atrophy. The significantly induced mRNA expressions of muscle ring finger 1 (MuRF1; gene name *Trim63*) and muscle atrophy F-box (Atrogin-1; gene name *Fbxo32*) were attenuated in RCE-treated atrophic L6 myotubes (Figure 2B). The marked decrease of phosphorylated Foxo3a, the transcription factor that activates the ubiquitin-proteasome system, in atrophy was restored by RCE (Figure 2C).

### 3.3. RCE Upregulated Protein Synthesis in TNF-α-Induced L6 Myotubes

**Figure 3:**
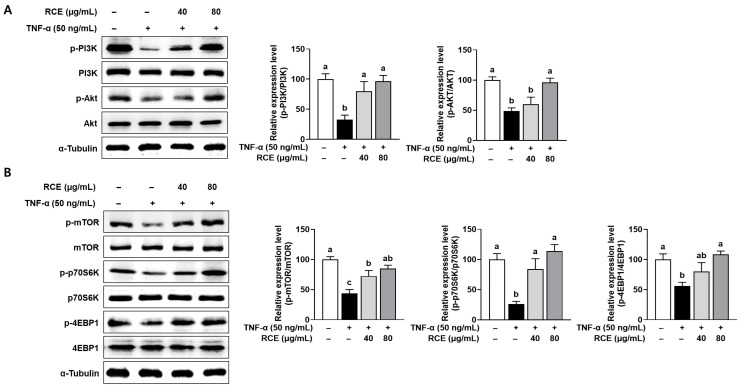
Effects of RCE on protein biosynthesis in TNF-α-stimulated L6 myotubes. The protein levels of (**A**) p-PI3K, PI3K, p-Akt, Akt, (**B**) p-mTOR, mTOR, p-p70S6K, p70S6K, p-4EBP1, and 4EBP1 in L6 myotubes were evaluated using Western blot with α-tubulin as a housekeeping gene. Group differences were assessed using one-way analysis of variance (ANOVA). Bars with different letters are significantly different (*p* < 0.05). Values are means ± SEM. RCE, *Rosa canina* extract; TNF-α, tumor necrosis factor-alpha; PI3K, phosphatidylinositol 3-kinase; p-PI3K, phosphorylated-PI3K; Akt, protein kinase B; p-Akt, phosphorylated-Akt; mTOR, mammalian target of rapamycin; p-mTOR, phosphorylated-mTOR; p70S6K, 70-kDa ribosomal S6 kinase; p-p70S6K, phosphorylated-p70S6K; 4EBP1, 4E-binding protein 1; p-4EBP1, phosphorylated-4EBP1.

Skeletal muscle mass is maintained by a dynamic balance between protein synthesis and protein degradation. Protein synthesis plays a central role in muscle growth, repair, and adaptation to physiological stimuli. The anabolic protein synthesis is tightly regulated by the PI3K/Akt/mTOR signaling pathway that acts as a critical node for integrating nutritional, hormonal, and mechanical signals [23]. To elucidate the molecular mechanisms underlying the anabolic effects of RCE, we examined the activation of the PI3K/Akt/mTOR signaling pathway, a key regulator of protein synthesis in skeletal muscles. TNF-α treatment markedly suppressed the phosphorylation levels of PI3K, Akt, mTOR, p70S6K, and 4EBP1 in atrophic L6 myotubes, indicating inhibition of anabolic signaling. However, treatment of RCE significantly restored the phosphorylation of these proteins (Figure 3A,B). Notably, p-mTOR levels were increased by 40.98% at 80 μg/mL RCE compared to the TNF-α-treated group. These findings suggest that RCE promotes muscle protein synthesis by activating the PI3K/Akt/mTOR signaling pathway, thereby partially contributing to muscle growth under inflammatory conditions.

### 3.4. Effects of RCE on Muscle Mass and Exercise Performance in Immobilized Mice

**Figure 4:**
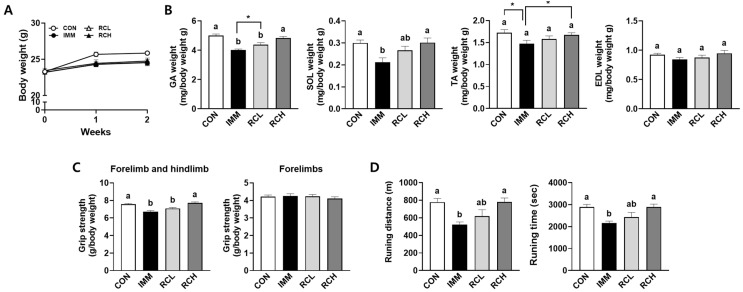
Effects of RCE on muscle weight and exercise performance in immobilized mice. (**A**) Body weights were measured. (**B**) The gastrocnemius (GA), soleus (SOL), tibialis anterior (TA), and extensor digitorum longus (EDL) muscle weights were measured. (**C**) Grip strengths of forelimb/hindlimb and forelimbs were evaluated using a grip strength meter. (**D**) Exercise capacity was measured using a treadmill test. *n* = 8–10 per group. Group differences were assessed using one-way analysis of variance (ANOVA). Bars with different letters are significantly different (*p* < 0.05). Asterisks (*) indicate statistically significant differences between the two groups based on unpaired *t*-test results (*p*  <  0.05). Values are means ± SEM. CON, mice received no surgery and were supplemented with saline; IMM, mice received immobilization surgery and were supplemented with saline; RCL, mice received immobilization surgery and were orally administered RCE at 200 mg/kg/day; RCH, mice received immobilization surgery and were orally administered RCE at 400 mg/kg/day.

Changes in GA, SOL, TA, and EDL muscle weight can serve as reliable biomarkers of immobilization-induced atrophy. Muscle weight is strongly correlated to exercise performance, one of the indicators of muscle strength [24]. To evaluate the effects of RCE on muscle atrophy in mice, we first measured the weight of different muscle types in immobilized C57BL/6N mice. Mice treated with immobilization showed decreased body weight compared with the CON group, but there was no significant difference in the RCE-treated groups (Figure 4A). Immobilization-induced atrophy significantly decreased the weights of GA, SOL, and TA in the IMM group, whereas the decreased muscle weights were recovered in the RCH group (Figure 4B). Next, muscle strength and endurance in immobilized mice were investigated by evaluating exercise performance parameters, including grip strength and treadmill-based running capacity. Immobilization of the hindlimb in the IMM group significantly reduced relative fore/hindlimb grip strength by 15.49% compared to the CON. In contrast, RCE administration markedly improved grip strength, with increases of 5.58% and 12.50% observed in the RCL and RCH groups, respectively. However, no significant differences were observed among groups in the relative grip strength of the forelimb (Figure 4C). Furthermore, both running distance and time to exhaustion were significantly reduced in the IMM group compared to the CON. RCE administration at the higher dose, RCH group, significantly improved both running distance and time, whereas the lower dose, RCL group, did not elicit a significant improvement (Figure 4D). Collectively, these findings indicate that RCE enhances hindlimb muscle strength and improves endurance capacity in mice with immobilization-induced muscle atrophy, suggesting a potential role for RCE in improving exercise performance under muscle-disuse conditions.

### 3.5. RCE Ameliorated the Phenotype Changes in the Muscle of Immobilized Mice

**Figure 5:**
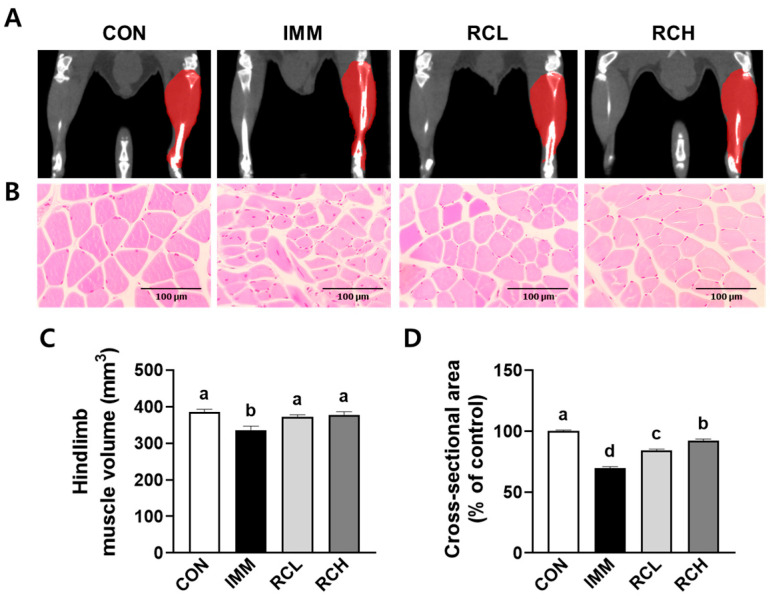
Effects of RCE on phenotype changes in the muscle of immobilized mice. (**A**) The right hindlimb muscle volumes of mice were measured using a micro-CT machine. (**B**) A cross-sectional area in the tibialis anterior muscle was evaluated using histological analysis. (**C**) Hindlimb muscle volume was quantified. (**D**) Cross-sectional area was quantified. *n* = 4–7 per group. Group differences were assessed using one-way analysis of variance (ANOVA). Bars with different letters are significantly different (*p* < 0.05). Values are means ± SEM. CON, mice received no surgery and were supplemented with saline; IMM, mice received immobilization surgery and were supplemented with saline; RCL, mice received immobilization surgery and were orally administered RCE at 200 mg/kg/day; RCH, mice received immobilization surgery and were orally administered RCE at 400 mg/kg/day.

The dominant fiber types of each muscle, including GA, SOL, TA, and EDL, lead to different functions [25]. To evaluate the effects of RCE on switching in muscle atrophy in mice, the phenotype changes in the muscle were assessed using Micro-CT imaging, quantification of the right hindlimb muscle weight, and histological analysis. Micro-CT analysis revealed that the right hindlimb muscle volumes of mice in the CON, IMM, RCL, and RCH groups were 385.98 ± 13.03 mm^3^, 336.08 ± 21.64 mm^3^, 371.68 ± 11.83 mm^3^, and 376.88 ± 17.79 mm^3^, respectively (Figure 5A,C). Muscle volume was significantly reduced in the IMM group compared to the CON group; however, RCE treatment restored muscle volume toward control levels in both the RCL and RCH groups. Furthermore, the myofiber cross-sectional area (CSA) of the TA muscle, which was reduced following immobilization, was significantly increased in the RCE-supplemented groups (Figure 5B,D). Collectively, these results demonstrate that RCE mitigates immobilization-induced muscle atrophy by enhancing muscle volume, mass, and fiber CSA.

### 3.6. RCE Attenuated Inflammation and Oxidative Stress in the Muscle of Immobilized Mice

**Figure 6:**
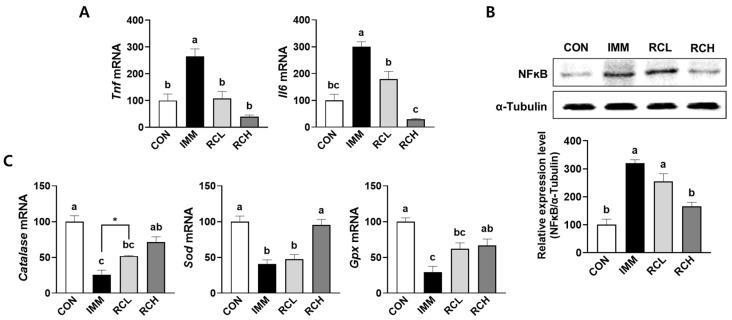
Effects of RCE on inflammation and oxidative stress in the tibialis anterior muscle of immobilized mice. (**A**) The mRNA levels of *Tnf* and *Il6* in the tibialis anterior muscle were evaluated using RT-PCR with β-actin as a housekeeping gene. (**B**) The protein levels of NF-κB in tibialis anterior muscle were evaluated using a Western blot with α-tubulin as a housekeeping gene. (**C**) The mRNA levels of *catalase*, *Sod*, and *Gpx* in the tibialis anterior muscle were evaluated using RT-PCR with β-actin as a housekeeping gene. *n* = 3–6 per group. Data are expressed as the mean ± SEM (% control) of individual experiments. Group differences were assessed using one-way analysis of variance (ANOVA). Bars with different letters are significantly different (*p* < 0.05). Asterisks (*) indicate statistically significant differences between the two groups based on unpaired *t*-test results (*p*  <  0.05). Values are means ± SEM. CON, mice received no surgery and were supplemented with saline; IMM, mice received immobilization surgery and were supplemented with saline; RCL, mice received immobilization surgery and were orally administered RCE at 200 mg/kg/day; RCH, mice received immobilization surgery and were orally administered RCE at 400 mg/kg/day; *Tnf*, gene name of tumor necrosis factor-alpha; Il6, gene name of interleukin 6; NFκB, nuclear factor kappa B; *Sod*, gene name of superoxide dismutase; *Gpx*, gene name of glutathione peroxidase.

Inflammation and oxidative stress are key mediators in the progression of muscle atrophy [20]. We measured the genes involved in these pathways in atrophic muscles induced by immobilization. The immobilization significantly induced the mRNA expressions of *Tnf* and *Il6* in the muscle of IMM groups, whereas the induction was downregulated in both RCL and RCH groups (Figure 6A). The significant upregulation of NF-κB protein level was observed in the muscle of the IMM group. In contrast, the marked induction of NF-κB protein was abolished in the atrophic muscle of the RCH group (Figure 6B). Furthermore, the mRNA expressions of *catalase*, *Sod*, and *Gpx* were significantly downregulated in the IMM group when compared with the CON group, whereas RCE treatment increased the mRNA expression of each gene (Figure 6C). These results suggest that RCE mitigates the inflammatory response and oxidative stress by modulating the signaling pathway involved in muscle atrophy induced by immobilization.

### 3.7. RCE Inhibited Protein Degradation in the Atrophic Muscle of Immobilized Mice

**Figure 7:**
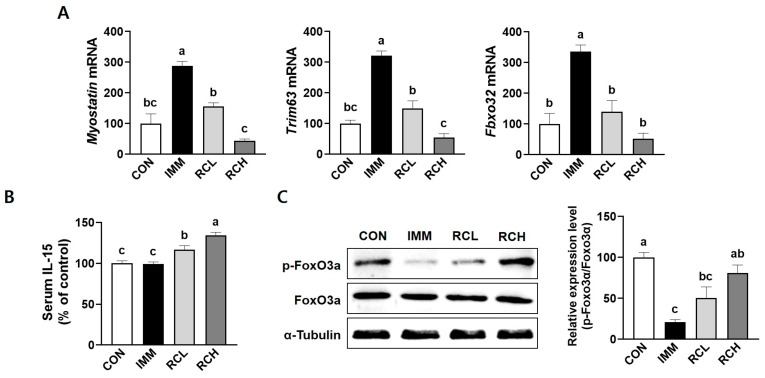
Effects of RCE on protein degradation-related pathways in tibialis anterior muscle. The protein levels of (**A**) The mRNA levels of myostatin, *Trim63*, and *Fbxo32* in tibialis anterior muscle were evaluated using RT-PCR with β-actin as a housekeeping gene. (**B**) Serum IL-15 levels were evaluated by an ELISA kit. (**C**) The protein levels of FoxO3a in the tibialis anterior muscle were evaluated using a Western blot with α-tubulin as a housekeeping gene. *n* = 3–6 per group. Data are expressed as the mean ± SEM of individual experiments. Group differences were assessed using one-way analysis of variance (ANOVA). Bars with different letters are significantly different (*p* < 0.05). CON, mice received no surgery and were supplemented with saline; IMM, mice received immobilization surgery and were supplemented with saline; RCL, mice received immobilization surgery and were orally administered RCE at 200 mg/kg/day; RCH, mice received immobilization surgery and were orally administered RCE at 400 mg/kg/day; *Trim63*, gene name of muscle ring finger 1; *Fbxo32*, gene name of muscle atrophy F-box; IL-15, interleukin 15; FoxO3a, forkhead box O3a; p-FoxO3a, phosphorylated FoxO3a.

Muscle atrophy is predominantly driven by the activation of specific proteolytic pathways, which accelerate protein catabolism and contribute to the progressive loss of muscle mass [22]. The mRNA expression of myostatin was significantly increased in the IMM group, but this increase was suppressed in the RCL and RCH groups (Figure 7A). The serum levels of IL-5, a myokine implicated in regulating muscle mass, were increased in the RCE-administered groups, RCL and RCH (Figure 7C). Likewise, the mRNA expression of the *Trim63* and *Fbxo32* was upregulated in the IMM group, compared with the CON group. However, the mRNA expression of each gene was significantly decreased following the RCE treatments (Figure 7C). Overall, these data suggest that RCE may alleviate proteolysis by downregulating genes involved in protein degradation.

### 3.8. RCE Upregulated Protein Synthesis-Related Pathway in the Muscle of Immobilized Mice

**Figure 8:**
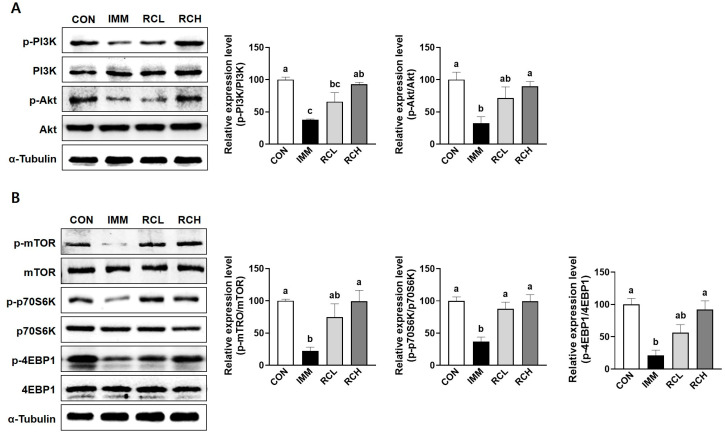
Effects of RCE on protein synthesis in tibialis anterior muscle. The protein levels of (**A**) p-PI3K, PI3K, p-Akt, Akt, (**B**) p-mTOR, mTOR, p-p70S6K, p70S6K, p-4EBP1, and 4EBP1 in tibialis anterior muscle were evaluated using Western blot with α-tubulin as a housekeeping protein. Data are expressed as the mean ± SEM (% control) of individual experiments. *n* = 3 per group. Group differences were assessed using one-way analysis of variance (ANOVA). Bars with different letters are significantly different (*p* < 0.05). CON, mice received no surgery and were supplemented with saline; IMM, mice received immobilization surgery and were supplemented with saline; RCL, mice received immobilization surgery and were orally administered RCE at 200 mg/kg/day; RCH, mice received immobilization surgery and were orally administered RCE at 400 mg/kg/day; PI3K, phosphatidylinositol 3-kinase; p-PI3K, phosphorylated-PI3K; Akt, protein kinase B; p-Akt, phosphorylated-Akt; mTOR, mammalian target of rapamycin; p-mTOR, phosphorylated-mTOR; p70S6K, 70-kDa ribosomal S6 kinase; p-p70S6K, phosphorylated-p70S6K; 4EBP1, 4E-binding protein 1; p-4EBP1, phosphorylated-4EBP1.

Reduced protein synthesis plays a central role in muscle atrophy, as it disrupts the balance of protein turnover in muscle and contributes to net muscle loss. In the IMM group, the phosphorylation of PI3K and Akt was significantly decreased compared to the CON group. However, treatment with RCE recovered these changes (Figure 8A). The oral administration of RCE reversed the decreased phosphorylation of mTOR, p70S6K, and 4EBP1 induced by immobilization (Figure 8B). Taken together, these results support the concept that RCE activates the PI3K/Akt/mTOR pathway.

## 4. Discussion

Skeletal muscle plays a critical role not only in locomotion but also in systemic metabolic homeostasis [26]. As one of the largest organs in the body by mass, skeletal muscle is a major site for glucose uptake, fatty acid oxidation, and amino acid storage, consequently contributing to energy homeostasis and metabolic regulation [27]. As it plays a central role in maintaining metabolic homeostasis, muscle atrophy, characterized by a loss of muscle mass and function, is increasingly recognized as a critical factor contributing to metabolic dysregulation. Numerous studies have demonstrated that reduced skeletal muscle mass is closely associated with insulin resistance, impaired glucose tolerance, and an elevated risk of developing metabolic disorders such as type 2 diabetes and metabolic syndrome [28]. The decline in muscle mass not only compromises physical function but also disrupts whole-body energy metabolism. Given this strong association, muscle atrophy is garnering increasing attention as both a marker and a potential therapeutic target in the prevention and management of metabolic disease [29,30]. Despite the multifactorial nature of muscle atrophy, common underlying mechanisms include increased oxidative stress, chronic inflammation, impaired myogenesis, and an imbalance between protein synthesis and degradation pathways [2,3]. Therefore, identifying therapeutic strategies that target these pathways to preserve muscle mass and function is of significant interest. In the present study, we investigated the potential of *Rosa canina*, a plant traditionally known for its antioxidant and anti-inflammatory properties, in attenuating muscle atrophy both in vitro and in vivo. Using L6 myotubes and an immobilized mouse model, we examined the effects of RCE on key signaling pathways involved in muscle inflammation, oxidative stress response, myogenic differentiation, protein synthesis, and proteolysis.

Chronic inflammation and oxidative stress are widely recognized as key contributors to the pathogenesis of muscle atrophy, primarily through the disruption of muscle protein turnover and the activation of catabolic signaling pathways [31]. In the present study, we demonstrated that RCE exerts potent anti-inflammatory and antioxidant effects in both TNF-α-stimulated L6 myotubes and an immobilization-induced muscle atrophy model in mice. RCE significantly downregulated the expression of key pro-inflammatory cytokines, including TNF-α and IL-6, and inhibited the activation of the NF-κB signaling pathway, a master regulator of inflammation known to promote proteolysis and inhibit myogenesis under catabolic conditions. The antioxidant properties of RCE have been well established in previous studies using various analytical methods, which demonstrated its strong radical-scavenging and redox activities [32,33]. In the present study, we evaluated the effects of RCE on antioxidant-related gene expression in both in vitro and in vivo muscle atrophy models, with the primary aim of elucidating the molecular mechanisms by which RCE exerts its protective effects via redox-related signaling pathways. Concurrently, RCE restored the expression of major antioxidant enzymes, i.e., catalase, SOD, and GPx, which were suppressed in response to TNF-α stimulation and immobilization. The dual modulation of inflammatory and oxidative stress pathways suggests that RCE may preserve muscle integrity by attenuating the feed-forward cycle of inflammation and oxidative stress that accelerates muscle mass loss. These findings are consistent with previous reports that have highlighted the strong antioxidant and anti-inflammatory properties of *Rosa canina*. Several studies have reported that *Rosa canina* reduces systemic inflammatory markers, such as C-reactive protein and IL-6, in clinical and preclinical models of chronic inflammation [34]. Moreover, several studies have demonstrated that *Rosa canina* supplementation enhances antioxidant defense systems in response to oxidative damage [13,14]. Although prior research has largely focused on systemic or joint inflammation, our study extends these observations to skeletal muscle, providing mechanistic evidence that supports the use of Rosa canina in conditions characterized by muscle wasting. Together, these data support the therapeutic potential of RCE as a natural intervention for muscle atrophy driven by inflammation and oxidative stress.

The maintenance of muscle mass is intricately regulated by myogenesis, which involves the activation of myogenic regulatory factors such as MyoD, myogenin and structural proteins like Myh [35]. In the present study, TNF-α suppressed the expression of these myogenic markers, contributing to impaired differentiation and atrophic conditions in L6 myotubes. In contrast, RCE treatment significantly restored the expression of these genes. These results suggest that RCE plays a protective role in preserving myogenic potential under inflammatory stress. Myostatin, a negative regulator of muscle growth, indirectly promotes muscle protein degradation by enhancing the transcription of atrophy-related genes, particularly those encoding ubiquitin E3 ligases, and inhibits protein synthesis via suppression of the Akt/mTOR pathway, a key driver of anabolic signaling [36]. The suppression of myostatin in atrophic L6 cells and muscle of immobilized mice by RCE further supports the anabolic effects of RCE on muscle development. Similar to these results, several studies have reported that flavonoids and phenolic acids rich in RCE enhance muscle differentiation and regeneration in various models. Quercetin, one of the major flavonoids found in *Rosa canina*, has been shown to promote myogenic differentiation by upregulating MyoD and myogenin expression in human skeletal muscle satellite cells [37]. Gallic acid increased myogenic gene expressions in mouse primary myoblasts [38]. These results collectively suggest that RCE preserves muscle regeneration potential through the synergistic actions of its polyphenols and antioxidants, offering protection against inflammation-induced impairments in myogenesis.

Muscle wasting is also driven by increased protein degradation through activation of the ubiquitin–proteasome system, particularly via E3 ubiquitin ligases MuRF1 and atrogin-1 [39]. In our study, both in vitro and in vivo atrophic conditions led to the upregulation of these genes, consistent with previous models of disuse atrophy. RCE treatment significantly downregulated the expression of these genes, suggesting a protective effect against excessive proteolysis. Notably, we also observed restoration of phosphorylated FoxO3a levels in L6 myotubes, indicating the role of RCE in suppressing the transcriptional activation of catabolic genes via the FoxO signaling axis. Parallel with our findings, quercetin has been shown to attenuate muscle loss by downregulating the expression of MuRF1 and Atrogin-1 in high-fat diet-induced obese mice [40]. IL-15 is a myokine, a cytokine derived from skeletal muscle, that plays a significant role in regulating muscle mass, mitochondrial biogenesis, and anti-inflammatory responses [41]. It has been implicated as a myokine that promotes muscle anabolism, inhibits muscle protein degradation, and plays a critical role in muscle remodeling. In the present study, we observed that plasma IL-15 levels were increased following RCE administration, despite no significant change in the immobilized (IMM) group. These results suggest that IL-15 may not play a central role in the early phase of immobilization-induced atrophy. In contrast, the observed elevation of IL-5 in RCE-treated groups may indicate a novel role for this cytokine in modulating muscle mass or immune-muscle interactions, although further exploration is required.

Anabolic signaling through the PI3K/Akt/mTOR pathway is crucial for sustaining protein synthesis and facilitating muscle hypertrophy [42]. Consistent with previous findings that TNF-α suppresses this pathway, we observed reduced phosphorylation of PI3K, Akt, mTOR, p70S6K, and 4EBP1 in atrophic L6 myotubes. RCE treatment reversed these changes and restored phosphorylation levels in a dose-dependent manner, highlighting its role in promoting anabolic signaling. These in vitro results were mirrored in vivo, where immobilized mice exhibited suppressed PI3K/Akt/mTOR pathway activity, which was significantly rescued by oral RCE administration. Consistent with these results, resveratrol regulated AKT/mTOR/Foxo signaling pathway in TNF-α-induced muscle atrophy in C2C12 cells [43].

Muscle weight and fiber CSA are key morphological indicators of muscle health, reflecting the net outcome of muscle protein turnover, regenerative capacity, and cellular integrity [44]. In the mouse immobilization model, RCE supplementation mitigated muscle mass loss in multiple hindlimb muscles. Histological analyses revealed that immobilization-induced muscle fiber atrophy, evidenced by reduced CSA, was alleviated by RCE treatment. The observed increase in muscle mass was accompanied by improved exercise performance, including grip strength and endurance, which are functional indicators of muscle strength and quality. Notably, the improvements in treadmill running distance and time to exhaustion in the RCH group further underscore the functional benefit of RCE supplementation in combating disuse-induced physical decline. These findings were supported by micro-CT analysis, which showed restoration of hindlimb muscle volume in RCE-treated mice.

The investigation of natural products should include a comprehensive evaluation of their biological efficacy, systemic safety, and potential toxicological effects in vivo. Previous studies have shown that oral administration of *R. canina* at 250–500 mg/kg/day effectively alleviated oxidative stress and neuroinflammation [17], while doses of 200–400 mg/kg/day protected against dextran sulfate sodium-induced colonic lesions through redox modulation and regulation of ion homeostasis [11]. Furthermore, acute toxicity assessments have indicated that *R. canina* doses up to 3200 mg/kg/day were well tolerated, with no observable adverse or toxicological manifestations. In line with previous findings, no mortality, behavioral abnormalities, or apparent pathological changes were observed in the present study throughout the two-week experimental period. To further verify systemic safety, liver and spleen weights were measured at the end of the study. No significant differences were observed between the RCE-treated and control groups, suggesting that RCE administration did not induce hepatic or splenic adverse effects. Collectively, these results support that the RCE doses employed in this study were within a physiologically safe range and sufficient to confer protective effects against muscle atrophy without adverse outcomes. Furthermore, the evaluation of the biological properties of a natural product must consider the potential contribution of its bioactive components. We conducted a qualitative analysis to identify major bioactive compounds in RCE and confirmed the presence of quinic acid. The quinic acid content in dried *Rosa Canina* was 0.1% confirmed by the HPLC chromatogram of RCE. Consistent with our findings, previous literature reported the quinic acid content in RCE is approximately 0.11% (*w*/*w*) [32]. These findings suggest that quinic acid may contribute to the observed anti-muscle atrophy effects of RCE. However, detailed compositional data were not included, as the primary objective of the present study was to evaluate the overall anti-muscle atrophy effects of RCE. Further studies are warranted to determine whether the protective effects of RCE on muscle atrophy are attributed to quinic acid.

## 5. Conclusions

Taken together, our results demonstrate that RCE counteracts muscle atrophy by modulating key regulatory pathways involved in inflammation, oxidative stress, myogenesis, protein synthesis, and degradation in atrophic L6 myotubes and immobilized mice. These findings provide mechanistic insights into the bioactivity of Rosa canina and support its potential application in mitigating muscle-wasting conditions. In conclusion, the present study supports that *Rosa canina* may be a promising natural therapeutic for preserving muscle health and metabolic homeostasis.

## Data Availability

The original contributions presented in the study are included in the article; further inquiries can be directed to the corresponding authors.

## Abbreviations

The following abbreviations are used in this manuscript:

TNF-α	tumor necrosis factor-alpha
IL	interleukin
IGF-1	insulin-like growth factor 1
PI3K	phosphatidylinositol 3-kinase
Akt	protein kinase B
mTOR	mammalian target of rapamycin
ROS	reactive oxygen species
NF-κB	nuclear factor kappa B
FoxO3a	forkhead box O3a (FoxO3a)
RCE	*Rosa canina* extract
p-PI3K	phosphorylated-PI3K
p-Akt	phosphorylated-Akt
p-mTOR	phosphorylated-mTOR
p70S6K	70-kDa ribosomal S6 kinase
p-p70S6K	phosphorylated-p70S6K
4EBP1	4E-binding protein 1
p-4EBP1	phosphorylated-4EBP1
DMEM	Dulbecco’s modified Eagle’s medium
FBS	fetal bovine serum
CON	mice received no surgery and were supplemented with saline
IMM	mice received immobilization surgery and were supplemented with saline
RCL	mice received immobilization surgery and were orally administered RCE at 200 mg/kg/day
RCH	mice received immobilization surgery and were orally administered RCE at 400 mg/kg/day
GA	gastrocnemius
SOL	soleus
TA	tibialis anterior
EDL	extensor digitorum longus
H&E	hematoxylin & eosin
ELISA	Enzyme-linked immunosorbent assay
RT-PCR	Reverse transcription-polymerase chain reaction
SDS-PAGE	sulfate-polyacrylamide gel electrophoresis
TBS-T	Tris-buffered saline containing 0.1% Tween 20
ECL	enhanced chemiluminescence
SEM	standard error of the mean
ANOVA	one-way analysis of variance
SOD	superoxide dismutase
GPX	glutathione peroxidase
MTT	thiazolyl blue tetrazolium bromide
MyoD	myogenic differentiation 1
MHC	myosin heavy chain
MuRF1	muscle ring finger 1
Atrogin-1	muscle atrophy F-box
CSA	cross-sectional area
